# Hypertriglyceridemia with Acute Pancreatitis in Pediatric Diabetic Ketoacidosis: A Case Report

**DOI:** 10.7759/cureus.3844

**Published:** 2019-01-08

**Authors:** Priyank J Yagnik, Pooja H Desai, Vinai M Modem

**Affiliations:** 1 Pediatrics, University of Kansas School of Medicine - Wichita, Wichita, USA; 2 Pediatrics, University of Texas Health Science Center at Houston, Frisco, USA

**Keywords:** diabetic ketoacidosis complications, hyperlipidemia, milky serum, plasmapheresis, abdominal pain, dka

## Abstract

A 16-year-old female with new-onset diabetic ketoacidosis (DKA) developed acute pancreatitis and hypertriglyceridemia within 24 hours after admission. Her insulin regimen was continued after resolution of DKA, and her pancreatitis with hypertriglyceridemia showed resolution. We are presenting a case of pediatric DKA with hypertriglyceridemia and pancreatitis treated with extended insulin.

## Introduction

Diabetic Ketoacidosis (DKA) is one of the most common diagnoses in Pediatric Intensive Care Unit (PICU). The most fatal complication of DKA is cerebral edema, but other non-fatal complications such as hypertriglyceridemia and pancreatitis are very rare and not commonly encountered in PICU [1-2]. We present the case of a new-onset DKA complicated by hypertriglyceridemia and acute pancreatitis.

## Case presentation

A previously healthy, non-obese, 16-year-old African-American female presented to a local hospital because of difficulty in breathing. Two weeks prior to this, she was diagnosed with oral thrush and prescribed nystatin by her primary care physician. She also had polyuria, polydipsia, weight loss, and decreased energy for a few days prior to presentation. She was found to have DKA at the local community hospital (serum glucose > 500 mg/dL, urine glucose > 1000 mg/dL, urine ketone > 80 mg/dL, capillary blood pH of 6.8, and serum sodium 110 mEq/L). She presented with moderate-to-severe dehydration and received two liters (~30 ml/kg) of crystalloid fluid bolus. She also received intravenous (IV) insulin bolus and sodium bicarbonate bolus before she was transferred to our tertiary care center.

**Figure 1:**
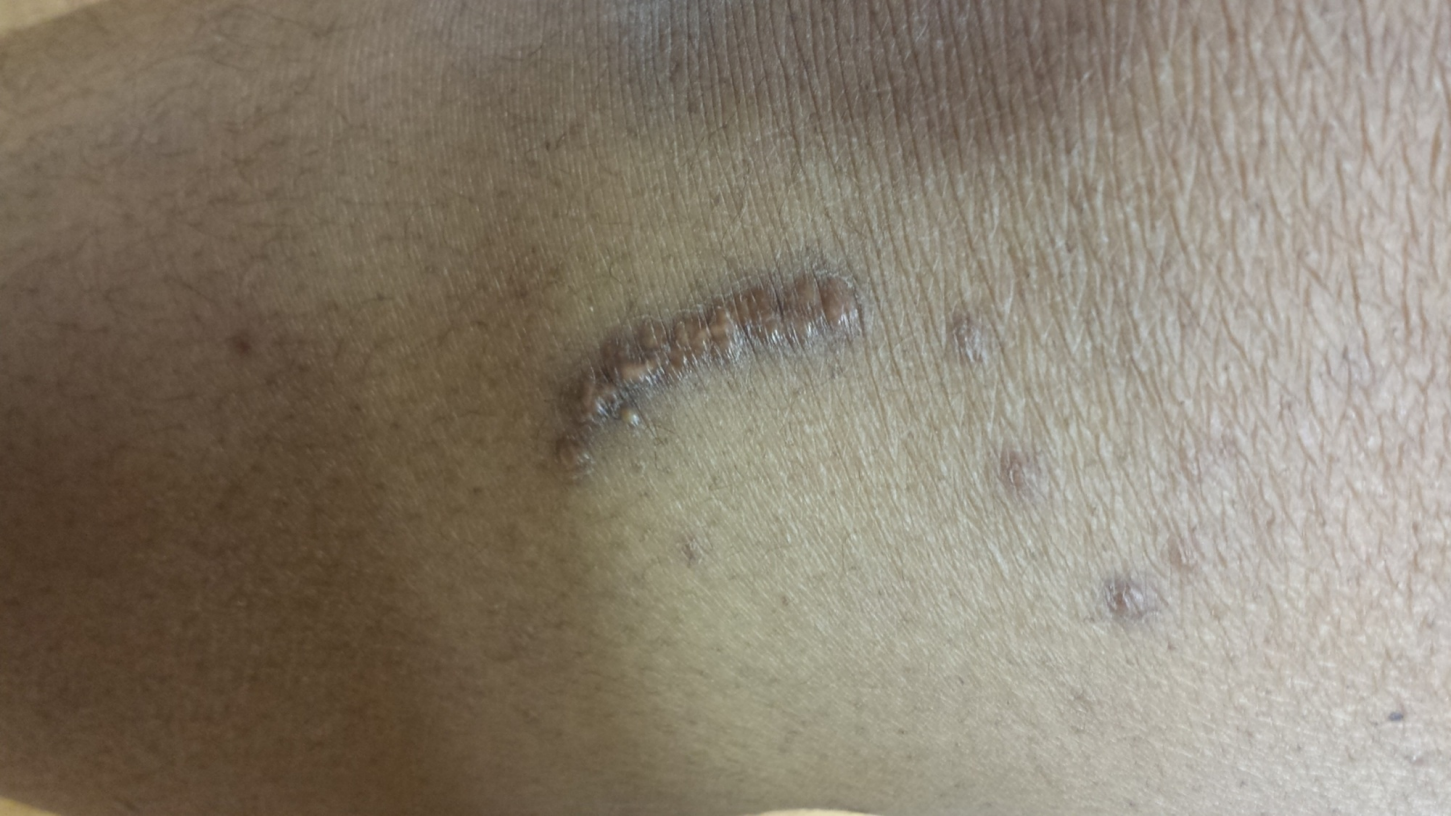
Skin lesions - multiple yellowish firm papules around the bilateral knees and scattered around the face and trunk

Upon arrival at the PICU, she continued to exhibit severe metabolic acidosis. Her examination was remarkable for Kussmaul breathing, altered mental status responding only to painful stimuli, and signs of poor perfusion (tachycardia up to 140/min, capillary refill time of 3-4 seconds, and bilateral weak peripheral pulses). She was also noted to have skin lesions, which were multiple yellowish firm papules, around both knees and scattered over the face and trunk, as seen in Figure 1. Point-of-care serum sodium level was 122 mEq/dL but laboratory could not perform any tests on the sample since it was "too thick". It was difficult to obtain a blood sample from the peripheral IV line and once obtained, it would turn "milky". Her serum sodium showed a downward trend despite improvement in her metabolic acidosis. The possibility of hyperlipidemia was considered based on the appearance of her blood sample. Her serum triglyceride (TG) level was found to be 930 mg/dL and total cholesterol 332 mg/dL.

She was continued on IV insulin drip and twice maintenance IV fluids. Her mental status improved eight to ten hours after the admission, along with stabilization of serum glucose, but developed diffused abdominal pain with anorexia 12 hours after getting admitted to PICU. Her serum amylase was 155 unit/L and lipase 1100 unit/L. Abdominal ultrasound was consistent with acute pancreatitis and ruled out acute appendicitis, gall stone, and pancreatic pseudocyst. Between 12 to 36 hours after admission, her highest serum TG level was noted as 2515 mg/dL, highest serum amylase as 612 unit/L, and highest serum lipase as 5387 unit/L. Due to pancreatitis and lack of appetite, she was continued on insulin drip and maintenance IV fluid even after resolution of her metabolic acidosis. Serum glucose was measured every two hours and there was no hypoglycemic episode. Serum levels of amylase, lipase, and TG were checked every 12 hours during her PICU admission. Approximately 48 hours after admission, her appetite improved along with downward trending of serum TG (614 mg/dL) and serum lipase (574 unit/L). She was started on fenofibrate for hyperlipidemia. Dermatology was consulted for skin lesions, and the pathology report confirmed xanthoma.

## Discussion

DKA is a life-threatening but preventable complication of type I diabetes mellitus. It is characterized by severe insulin deficit leading to hyperglycemia, hyperosmolar dehydration, and accumulation of ketones in the serum. Cerebral edema is encountered in about 1% cases of pediatric DKA but accounts for more than 20% mortality in DKA [1]. Hyperlipidemia and acute pancreatitis are also among the rare complications of DKA that are not life-threatening.

Hyperglycemia is the common etiology of hyponatremia in DKA patients. Due to the osmotic flux, hyperglycemia causes shift of water from intracellular compartment to extracellular compartment, leading to dilutional hyponatremia. The corrected serum sodium in such cases is calculated by the following equation: corrected sodium = measured sodium + [1.6 (glucose – 100)/100]. Moreover, hyperlipidemia causes displacement of water-containing sodium, leading to pseudohyponatremia. A more accurate sodium can be measured after ultracentrifugation of the sample [3].

The insulin deficiency in type I diabetes mellitus causes hypertriglyceridemia. Insulin deficiency decreases the activity of lipoprotein lipase, which is responsible for the conversion of triglyceride to fatty acid. The triad of DKA, hyperlipidemia (specifically hypertriglyceridemia), and acute pancreatitis have been well described in adult literature. In an adult study conducted by Nair et al., hypertriglyceridemia (> 500 mg/dl) and radiographic evidence of acute pancreatitis were found in 11% and 22% of the patients, respectively, when 100 adult patients with DKA were studied [4].

The association of DKA with hypertriglyceridemia and elevated serum amylase/lipase has also been described in the pediatric population. In a study published in 2004 involving 50 children with DKA, the authors found 40% of the patients presented with hypertriglyceridemia (> 200 mg/L), 38% with hyperamylasemia, and 19% with hyperlipasemia. Only one patient exhibited clinically significant acute pancreatitis [5]. Pancreatic enzyme elevation is common within 12-24 hours of treatment of pediatric DKA [2].

A review by Tsuang et al. described that a TG level of > 1000 mg/L is required to ascribe the causation of acute pancreatitis. Severe hypertriglyceridemia has been treated with insulin, heparin and/or plasmapheresis, but comparative trials are lacking. A consensus has been established to bring a serum TG level of < 500 mg/L for symptomatic relief. Insulin activates lipoprotein lipase, and heparin stimulates the release of lipoprotein lipase from endothelium, leading to TG degradation. Plasmapheresis is a symptomatic treatment that acutely decreases the serum level of TG, thereby reducing the viscosity of blood. Oral antihyperlipidemics are recommended in cases where patients can tolerate an oral diet. Among these diets, fibrates can lower TG by 40%-60% [6].

The first case report of diabetic hypertriglyceridemia (serum TG 7120 mg/dL) being successfully treated by plasmapheresis was described in 1978 [7]. Lutfi et al. described the first pediatric case of DKA with severe hypertriglyceridemia (serum TG > 16000 mg/dL) that was successfully treated with one course of plasmapheresis. In this case report, plasmapheresis was indicated not only for hypertriglyceridemia but also for worsening abdominal pain, acute kidney injury (serum creatinine 1.2 mg/dL), and development of pleural effusion. Plasmapheresis resulted in the significant improvement of abdominal pain along with a decrease in the serum TG level from > 5000 mg/L to 1000 mg/L. The patient was started on fenofibrate upon discharge as well [8].

The patient described in the current case report was continued on IV insulin drip upon admission to PICU and then administered subcutaneous insulin after her appetite improved, approximately 48 hours after admission. IV insulin drip was continued even after resolution of her metabolic acidosis. Her serum amylase, lipase, and TG levels started trending down with insulin therapy alone. After being transferred to the floor, she was started on fenofibrate for hypertriglyceridemia. A follow-up visit two weeks after this episode showed a significant reduction of TG level to 170mg/dl. Dermatology concluded her skin lesion to be xanthoma after the biopsy. At the time of this writing, the patient was being evaluated for familial hypercholesterolemia.

## Conclusions

Elevation of serum amylase and lipase are common in DKA patients. Clinically significant acute pancreatitis can be attributed to severe hypertriglyceridemia if the serum TG level is > 1000 mg/dL. Treatment options include insulin, heparin, plasmapheresis, and fibrinolytic agents.
